# Supplementary material for: Alternative ecological strategies lead to avian brain size bimodality in variable habitats
Source: Nat Commun. 2019 Aug 23;10:3818. doi: 10.1038/s41467-019-11757-x (PMC6707158; doi:10.1038/s41467-019-11757-x)
Supplement: Supplementary file 1 — Supplementary Information [file 41467_2019_11757_MOESM1_ESM.pdf]

Supplementary Information

Alternative ecological strategies lead to avian brain size bimodality in variable habitats

Fristoe and Botero

This PDF file includes:

Supplementary Figs. 1 to 4
Supplementary Tables 1 to 6
Supplementary References

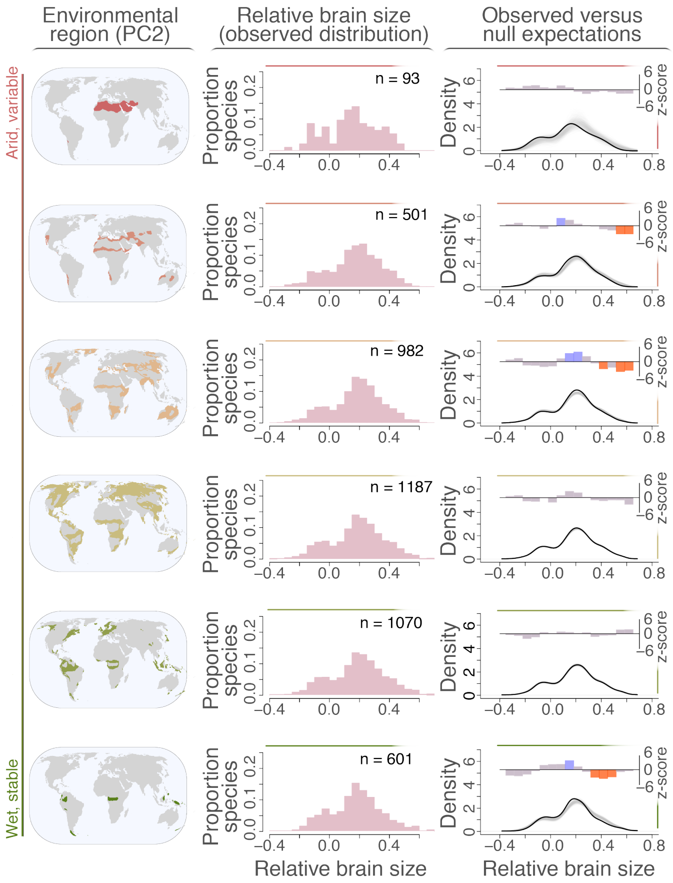

Supplementary Fig. 1. The global distribution of brain size for resident birds in relation to environmental PC2. The observed distributions of relative brain size for 1304 species of resident birds (second column) within environmental bins (first column) are compared to null expectations (third column). Maps represent regions along a composite environmental measure (PC2), with areas of low, seasonal, and unpredictable rainfall in red (low PC2 scores) and wet, stable areas in green (high PC2 scores)[1]. Relative brain size distributions include all species with breeding ranges that overlap with the given environmental region. The density estimation of relative brain sizes for the *n* species observed within the environmental region (solid black lines in third column) are compared to 10000 density estimations derived from *n* randomly sampled species (1000 examples plotted as light grey lines). The bars above density estimations depict the z-scores from comparisons between observed and null distributions, with significant over-representation of a given relative brain size in blue, significant under-representation in red, and no significant difference in grey (see methods and caption for fig. 1). Source data are provided as a Source Data file.

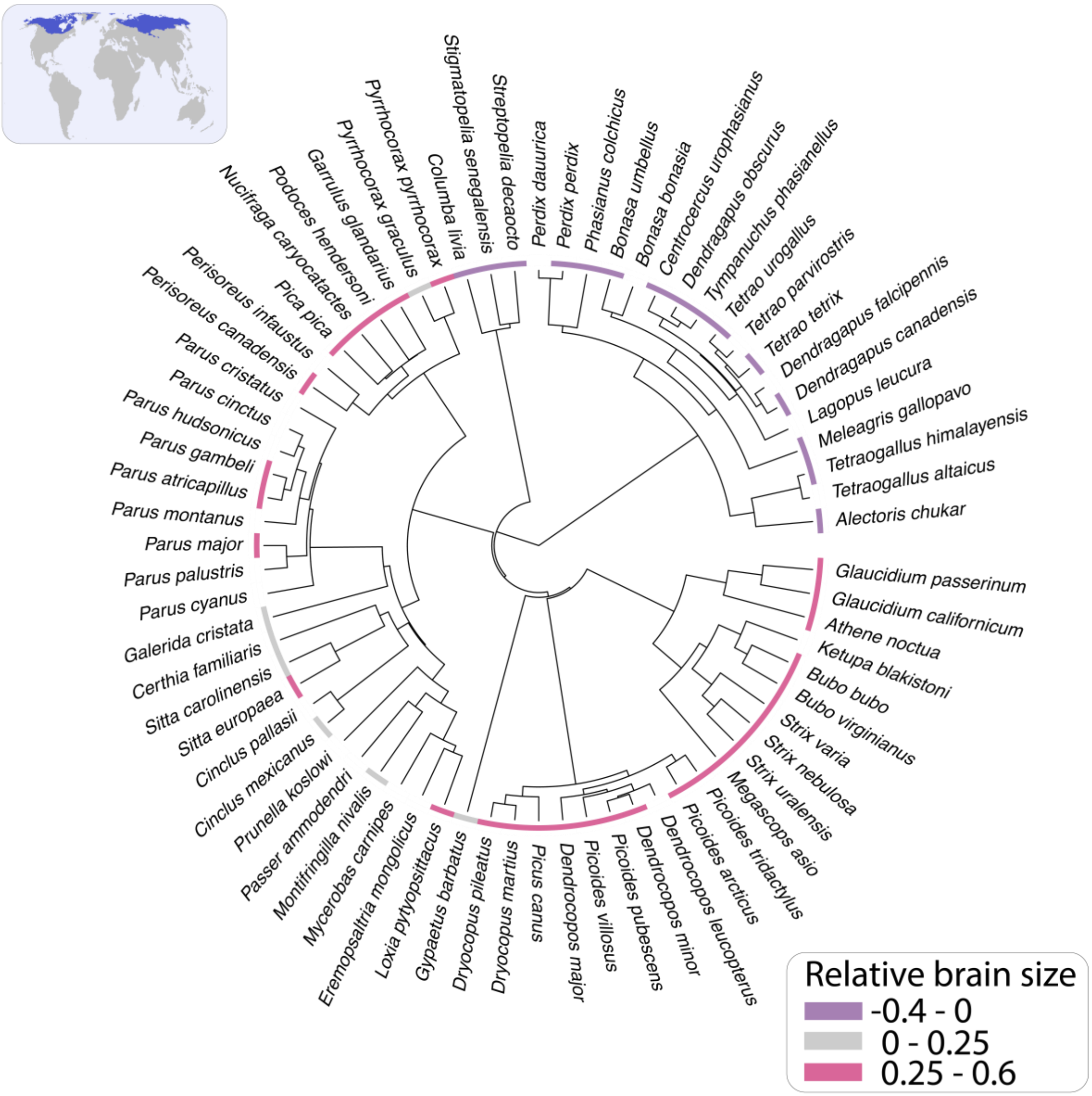

Supplementary Fig. 2. Phylogenetic distribution for all species occurring within the most variable environments. Species included are those that occur in regions with the lowest values of environmental PC1 (depicted on map in blue). Species with available data on relative brain size are categorized as small (purple), intermediate (grey), or large (pink). As relative brain size is phylogenetically conserved (1), missing data clustered within a particular clade could indicate whether certain brain sizes are under sampled. Tree is randomly selected from a posterior distribution constructed using the Hackett backbone from (2).

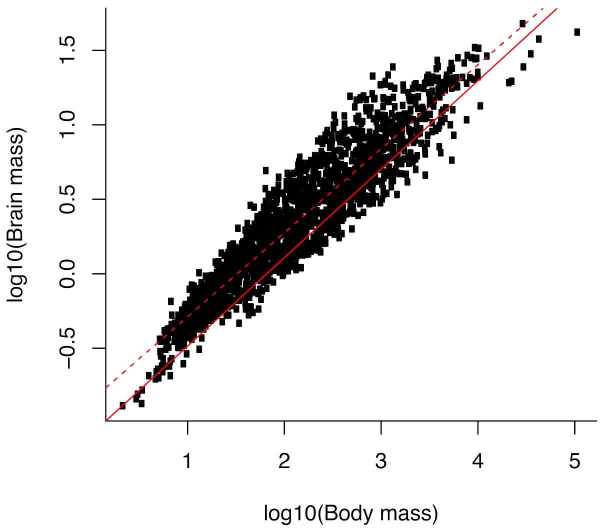

Supplementary Fig. 3. The allometric relationship between bran mass and body mass. Regression slopes from PGLS (solid red line; mean slope ± s.d = 0.594 ± 0.001) and OLS (dashed red line; slope ± SE = 0.563 ± 0.004) that are both below one indicate a sublinear relationship where the ratio of brain mass/body mass tends to decrease with increasing body mass. Source data are provided as a Source Data file.

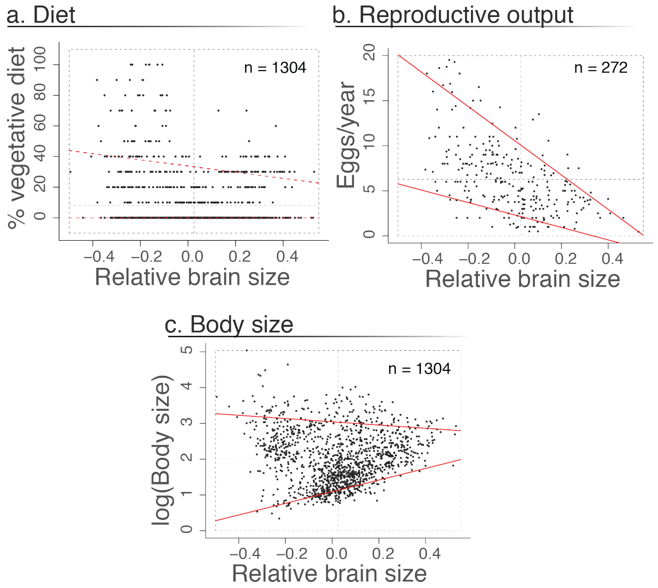

Supplementary Fig. 4. Constraints imposed by relative brain size on additional traits. The effects of relative brain size (here measured as residuals from ordinary least squares regression) on diet (a; upper quantile regression line: $\tau = 0.9$, $\beta \pm SE = -20.18 \pm 10.78$, $p = 0.06$; lower quantile regression line: $\tau = 0.1$, $\beta \pm SE = 0.00 \pm 0.00$, p = N/A), reproductive output (b; upper quantile regression line: $\tau = 0.9$, $\beta \pm SE = -19.04 \pm 1.76$, $p << 0.001$; lower quantile regression line: $\tau = 0.1$, $\beta \pm SE = -6.91 \pm 1.26$, $p << 0.001$), and body size (c; upper quantile regression line: $\tau = 0.9$, $\beta \pm SE = -0.45 \pm 0.15$, $p < 0.01$; lower quantile regression line: $\tau = 0.1$, $\beta \pm SE = 1.63 \pm 0.12$, $p << 0.001$) are tested in our global sample of resident species (see caption for fig. 3). Source data are provided as a Source Data file.

Supplementary Table 1. Summary of principal component analysis of global climatic variables. Loadings for main contributors to each component are in bold.

		Loadings	
**Variable**	**Transformation**	**PC1**	**PC2**
Temperature predictability	log	**0.96**	0.11
Temperature variance	boxcox	**-0.95**	-0.22
Mean temperature	inverse log	**0.91**	-0.23
Mean precipitation	boxcox	0.49	**0.78**
Precipitation coefficient of variation	boxcox	0.42	**-0.84**
Precipitation predictability	inverse log	-0.15	**0.93**
Cumulative variance		0.51	0.9

Supplementary Table 2. Summary statistics for comparisons between observed and expected frequencies of resident species of different relative brain sizes (columns; calculated as the residuals from ordinary least squares regression) in environmental regions spanning the range of environmental PC1 (rows). Low scores of PC1 represent cold, seasonal, and unpredictable environments (depicted in purple); high scores of PC1 represent warm, stable regions (depicted in green). Z-scores were computed by comparing the observed density estimate at a given brain size to the distribution of expected density estimates obtained through randomizations and the resulting p-values within an environmental region have been corrected for multiple comparisons. Cells highlighted in blue indicate brain sizes that area over-represented compared to null expectations in a given environment, those in red indicate brain sizes that are under-represented in an environment, and grey indicates brain sizes that do not significantly differ from null expectations in a given region. See methods for more details.

	Relative brain size														
Environmental PC1	-0.5 – -0.43	-0.43 – -0.36	-0.36 – -0.29	-0.29 – -0.22	-0.22 – -0.15	-0.15 – -0.08	-0.08 – -0.01	-0.01 – 0.05	0.05 – 0.12	0.12 – 0.19	0.19 – 0.26	0.26 – 0.33	0.33 – 0.4	0.4 – 0.47	0.47 – 0.54
-1.83 – -1.21	z = 5.32 p < 0.01	z = 3.83 p < 0.01	z = 1.97 p = 0.24	z = 0.1 p = 1.00	z = -1.37 p = 0.52	z = -2.75 p = 0.05	z = -3.73 p < 0.01	z = -3.33 p = 0.01	z = -2.4 p = 0.12	z = -0.18 p = 1.00	z = 2.32 p = 0.12	z = 3.78 p < 0.01	z = 4.02 p < 0.01	z = 3.17 p = 0.01	z = 1.9 p = 0.24
-1.21 – -0.59	z = 4.85 p < 0.01	z = 4.44 p < 0.01	z = 2.92 p = 0.03	z = 0.84 p = 1.00	z = -1.24 p = 0.86	z = -3.19 p = 0.01	z = -4.16 p < 0.01	z = -3.42 p = 0.01	z = -2.11 p = 0.21	z = 0.34 p = 1.00	z = 2.83 p = 0.03	z = 4.05 p < 0.01	z = 3.67 p < 0.01	z = 1.96 p = 0.25	z = 0.33 p = 1.00
-0.59 – 0.03	z = 2.62 p = 0.12	z = 3.03 p = 0.04	z = 1.42 p = 1.00	z = -0.87 p = 1.00	z = -0.38 p = 1.00	z = 1.41 p = 1.00	z = 0.25 p = 1.00	z = 0.25 p = 1.00	z = -0.51 p = 1.00	z = 0.18 p = 1.00	z = -0.49 p = 1.00	z = 0.2 p = 1.00	z = -0.58 p = 1.00	z = -2.41 p = 0.21	z = -2.38 p = 0.21
0.03 – 0.65	z = 0.71 p = 1.00	z = 0.53 p = 1.00	z = 0.01 p = 1.00	z = -0.16 p = 1.00	- z = 0.81 p = 1.00	z = 1.25 p = 1.00	z = 0.6 p = 1.00	z = 1.04 p = 1.00	z = 1.15 p = 1.00	z = 1.59 p = 1.00	z = 0.28 p = 1.00	z = -1.75 p = 0.97	z = -2.35 p = 0.24	z = -2.5 p = 0.17	z = -3.42 p = 0.01
0.65 – 1.27	z = -0.5 p = 1.00	z = -2.3 p = 0.18	z = -3.21 p = 0.02	z = -2 p = 0.32	z = -2.33 p = 0.18	z = 1.09 p = 1.00	z = 5.49 p < 0.01	z = 3.6 p < 0.01	z = 2.54 p = 0.11	z = 0.66 p = 1.00	z = -3 p = 0.03	z = -4.27 p < 0.01	z = -1.68 p = 0.56	z = -0.64 p = 1.00	z = -1.53 p = 0.63
1.27 – 1.89	z = -2.12 p = 0.34	z = -3.25 p = 0.01	z = -1.81 p = 0.64	z = 0.45 p = 1.00	z = 1.72 p = 0.68	z = 2.22 p = 0.32	z = 3.33 p = 0.01	z = 1.24 p = 1.00	z = 0.7 p = 1.00	z = -1.56 p = 0.84	z = -3.29 p = 0.01	z = -2.18 p = 0.32	z = -1.34 p = 1.00	z = -0.42 p = 1.00	z = 0.45 p = 1.00

Supplementary Table 3. Summary statistics for comparisons between observed and expected frequencies of migratory species of different relative brain sizes (columns; calculated as the residuals from ordinary least squares regression) in environmental regions spanning the range of environmental PC1 (rows). Low scores of PC1 represent cold, seasonal, and unpredictable environments (depicted in purple); high scores of PC1 represent warm, stable regions (depicted in green). Z-scores were computed by comparing the observed density estimate at a given brain size to the distribution of expected density estimates obtained through randomizations and the resulting p-values within an environmental region have been corrected for multiple comparisons. Cells highlighted in blue indicate brain sizes that area over-represented compared to null expectations in a given environment, those in red indicate brain sizes that are under-represented in an environment, and grey indicates brain sizes that do not significantly differ from null expectations in a given region. See methods for more details.

	Relative brain size														
Env. PC1	-0.5 – -0.43	-0.43 – -0.36	-0.36 – -0.29	-0.29 – -0.22	-0.22 – -0.15	-0.15 – -0.08	-0.08 – -0.01	-0.01 – 0.05	0.05 – 0.12	0.12 – 0.19	0.19 – 0.26	0.26 – 0.33	0.33 – 0.4	0.4 – 0.47	0.47 – 0.54
-1.83 – -0.59	z = 1.53 p = 1.00	z = 1.15 p = 1.00	z = -0.48 p = 1.00	z = -0.24 p = 1.00	z = 0.88 p = 1.00	z = 1.06 p = 1.00	z = -0.95 p = 1.00	z = -0.18 p = 1.00	z = 0.01 p = 1.00	z = -1.07 p = 1.00	z = 0.39 p = 1.00	z = 1.8 p = 0.93	z = -0.08 p = 1.00	z = -2.36 p = 0.25	z = -2.86 p = 0.06
0.65 – 1.89	z = -0.34 p = 1.00	z = 0.1 p = 1.00	z = 0.54 p = 1.00	z = 0.99 p = 1.00	z = -2.07 p = 0.46	z = -3.63 p < 0.01	z = 0.23 p = 1.00	z = 2.11 p = 0.45	z = 2.35 p = 0.26	z = 2.06 p = 0.46	z = -0.89 p = 1.00	z = -1.01 p = 1.00	z = 0.02 p = 1.00	z = 0.77 p = 1.00	z = 0.26 p = 1.00

Supplementary Table 4. Summary statistics for comparisons between observed and expected frequencies of resident species of different trait combinations (columns; combinations of small or large relative brain size with either low or high percentage of vegetative diet; relative brain size calculated as residuals from ordinary least squares regression) in environmental regions spanning the range of environmental PC1 (rows). Low scores of PC1 represent cold, seasonal, and unpredictable environments (depicted in purple); high scores of PC1 represent warm, stable regions (depicted in green). Z-scores were computed by comparing the observed number of species with a given trait combination to the distribution of species counts obtained through randomizations and the resulting p-values within an environmental region have been corrected for multiple comparisons. Cells highlighted in blue indicate trait combinations that area over-represented compared to null expectations in a given environment, those in red indicate trait combinations that are under-represented in an environment, and grey indicates trait combinations that do not significantly differ from null expectations in a given region. See methods for more details.

Environmental PC1	Small brain Low veg	Small brain High veg	Large brain Low veg	Large brain High veg
-1.83 – -1.21	z = -4.35; p < 0.01	z = 2.98; p = 0.01	z = 2.8; p = 0.01	z = -1.24; p = 0.22
-1.67 – -1.05	z = -4.89; p < 0.01	z = 2.6; p = 0.02	z = 3.59; p < 0.01	z = -1.16; p = 0.25
-1.5 – -0.88	z = -5.22; p < 0.01	z = 2.79; p = 0.01	z = 3.85; p < 0.01	z = -1.4; p = 0.16
-1.34 – -0.72	z = -5.36; p < 0.01	z = 3.59; p < 0.01	z = 3.3; p < 0.01	z = -1.27; p = 0.20
-1.18 – -0.56	z = -5.64; p < 0.01	z = 4.47; p < 0.01	z = 2.89; p = 0.01	z = -1.05; p = 0.29
-1.01 – -0.39	z = -5.02; p < 0.01	z = 5.1; p < 0.01	z = 2.29; p = 0.04	z = -1.7; p = 0.09
-0.85 – -0.23	z = -3.88; p < 0.01	z = 4.63; p < 0.01	z = 1.79; p = 0.07	z = -2.12; p = 0.07
-0.69 – -0.07	z = -4.09; p < 0.01	z = 5.6; p < 0.01	z = 1.27; p = 0.21	z = -2.13; p = 0.07
-0.52 – 0.1	z = -4.3; p < 0.01	z = 7.38; p < 0.01	z = -0.35; p = 0.73	z = -1.38; p = 0.34
-0.36 – 0.26	z = -3.93; p < 0.01	z = 5.65; p < 0.01	z = 0.25; p = 0.81	z = -0.83; p = 0.81
-0.2 – 0.42	z = -3.74; p < 0.01	z = 5.12; p < 0.01	z = 0.51; p = 0.76	z = -0.88; p = 0.76
-0.04 – 0.58	z = -3.1; p = 0.01	z = 4.42; p < 0.01	z = 0.33; p = 0.81	z = -0.83; p = 0.81
0.13 – 0.75	z = -2.11; p = 0.10	z = 3.83; p < 0.01	z = -0.23; p = 0.84	z = -0.81; p = 0.84
0.29 – 0.91	z = -1.39; p = 0.49	z = 2.32; p = 0.08	z = -0.24; p = 1.00	z = -0.19; p = 1.00
0.45 – 1.07	z = -0.66; p = 1.00	z = 2.45; p = 0.06	z = -0.61; p = 1.00	z = -0.79; p = 1.00
0.62 – 1.24	z = 0.71; p = 0.73	z = 1.64; p = 0.41	z = -1.12; p = 0.73	z = -1.16; p = 0.73
0.78 – 1.4	z = 3.7; p < 0.01	z = -2.14; p = 0.10	z = -0.88; p = 0.38	z = -1.69; p = 0.18
0.94 – 1.56	z = 5.72; < 0.01	z = -3.61; p < 0.01	z = -1.54; p = 0.12	z = -2.1; p = 0.07
1.11 – 1.73	z = 6.17; < 0.01	z = -3.83; p < 0.01	z = -1.38; p = 0.17	z = -2.88; p = 0.01
1.27 – 1.89	z = 5.81; < 0.01	z = -3.78; p < 0.01	z = -0.99; p = 0.32	z = -3.12; p < 0.01

Trait Combination

Supplementary Table 5. Summary statistics for comparisons between observed and expected frequencies of resident species with different trait combinations (columns; combinations of small or large relative brain size with either low or high annual reproductive output; relative brain size calculated as residuals from ordinary least squares regression) in environmental regions spanning the range of environmental PC1 (rows). Low scores of PC1 represent cold, seasonal, and unpredictable environments (depicted in purple); high scores of PC1 represent warm, stable regions (depicted in green). Z-scores were computed by comparing the observed number of species with a given trait combination to the distribution of species counts obtained through randomizations and the resulting p-values within an environmental region have been corrected for multiple comparisons. Cells highlighted in blue indicate trait combinations that area over-represented compared to null expectations in a given environment, those in red indicate trait combinations that are under-represented in an environment, and grey indicates trait combinations that do not significantly differ from null expectations in a given region. See methods for more details.

Trait Combination

Environmental PC1	Small brain Low eggs	Small brain High eggs	Large brain Low eggs	Large brain High eggs
-1.83 – -1.21	z = -2.9; p = 0.02	z = -0.04; p = 0.97	z = 1.08; p = 0.56	z = 1.94; p = 0.16
-1.67 – -1.05	z = -3.33; p < 0.01	z = -1; p = 0.51	z = 1.14; p = 0.51	z = 3.83; p < 0.01
-1.5 – -0.88	z = -3.53; p < 0.01	z = -1.24; p = 0.43	z = 0.95; p = 0.43	z = 4.91; p < 0.01
-1.34 – -0.72	z = -3.43; p < 0.01	z = -0.87; p = 0.77	z = 0.78; p = 0.77	z = 4.39; p < 0.01
-1.18 – -0.56	z = -3.36; p < 0.01	z = -0.19; p = 1.00	z = -0.03; p = 1.00	z = 4.49; p < 0.01
-1.01 – -0.39	z = -3.88; p < 0.01	z = 0.43; p = 1.00	z = 0.18; p = 1.00	z = 3.9; p < 0.01
-0.85 – -0.23	z = -3.69; p < 0.01	z = 0.44; p = 1.00	z = 0; p = 1.00	z = 3.95; p < 0.01
-0.69 – -0.07	z = -3.32; p < 0.01	z = 0.48; p = 1.00	z = 0.04; p = 1.00	z = 3.36; p < 0.01
-0.52 – 0.1	z = -3.14; p = 0.01	z = 1.57; p = 0.23	z = -1.17; p = 0.24	z = 3.43; p < 0.01
-0.36 – 0.26	z = -2.48; p = 0.04	z = 0.49; p = 1.00	z = -0.41; p = 1.00	z = 3.03; p = 0.01
-0.2 – 0.42	z = -2.37; p = 0.07	z = -0.69; p = 0.49	z = 1.17; p = 0.48	z = 2.1; p = 0.11
-0.04 – 0.58	z = -1.22; p = 0.67	z = -1.12; p = 0.67	z = 0.74; p = 0.67	z = 2.02; p = 0.17
0.13 – 0.75	z = 0.54; p = 1.00	z = -2; p = 0.18	z = 0.28; p = 1.00	z = 1.94; p = 0.18
0.29 – 0.91	z = 1.49; p = 0.41	z = -2.13; p = 0.13	z = 0.13; p = 0.89	z = 1.2; p = 0.46
0.45 – 1.07	z = 0.75; p = 1.00	z = -1.04; p = 1.00	z = 0.11; p = 1.00	z = 0.5; p = 1.00
0.62 – 1.24	z = 1.22; p = 0.84	z = -1.25; p = 0.84	z = -0.28; p = 0.84	z = 0.85; p = 0.84
0.78 – 1.4	z = 0.63; p = 1.00	z = -1.1; p = 1.00	z = 0.06; p = 1.00	z = 0.8; p = 1.00
0.94 – 1.56	z = 1.73; p = 0.33	z = -1.26; p = 0.63	z = -0.57; p = 0.99	z = 0.68; p = 0.99
1.11 – 1.73	z = 1.85; p = 0.26	z = -0.77; p = 1.00	z = -0.91; p = 1.00	z = 0.34; p = 1.00
1.27 – 1.89	z = 2.01; p = 0.18	z = -1.25; p = 0.64	z = 0.04; p = 1.00	z = -0.67; p = 1.00

Supplementary Table 6. Summary statistics for comparisons between observed and expected frequencies of resident species with different trait combinations (columns; combinations of small or large relative brain size with either small or large body size; relative brain size calculated as residuals from ordinary least squares regression) in environmental regions spanning the range of environmental PC1 (rows). Low scores of PC1 represent cold, seasonal, and unpredictable environments (depicted in purple); high scores of PC1 represent warm, stable regions (depicted in green). Z-scores were computed by comparing the observed number of species with a given trait combination to the distribution of species counts obtained through randomizations and the resulting p-values within an environmental region have been corrected for multiple comparisons. Cells highlighted in blue indicate trait combinations that area over-represented compared to null expectations in a given environment, those in red indicate trait combinations that are under-represented in an environment, and grey indicates trait combinations that do not significantly differ from null expectations in a given region. See methods for more details.

Environmental PC1	Small brain Small body	Small brain Large body	Large brain Small body	Large brain Large body
-1.83 – -1.21	z = -2.97; p = 0.01	z = 0.8; p = 0.85	z = 0.5; p = 0.85	z = 1.75; p = 0.24
-1.67 – -1.05	z = -3.19; p = 0.01	z = 0.01; p = 0.99	z = 0.86; p = 0.78	z = 2.35; p = 0.06
-1.5 – -0.88	z = -3.24; p < 0.01	z = -0.11; p = 0.91	z = 0.94; p = 0.69	z = 2.4; p = 0.05
-1.34 – -0.72	z = -3.35; p < 0.01	z = 0.53; p = 0.77	z = 0.87; p = 0.77	z = 1.99; p = 0.14
-1.18 – -0.56	z = -3.77; p < 0.01	z = 1.4; p = 0.32	z = 0.54; p = 0.59	z = 1.94; p = 0.16
-1.01 – -0.39	z = -4.19; p < 0.01	z = 2.98; p = 0.01	z = 0.19; p = 0.85	z = 1.18; p = 0.48
-0.85 – -0.23	z = -3.21; p = 0.01	z = 2.88; p = 0.01	z = -0.47; p = 0.69	z = 0.95; p = 0.69
-0.69 – -0.07	z = -3.22; p < 0.01	z = 3.49; p < 0.01	z = -0.19; p = 1.00	z = 0.1; p = 1.00
-0.52 – 0.1	z = -1.43; p = 0.46	z = 2.85; p = 0.02	z = -0.64; p = 0.89	z = -0.76; p = 0.89
-0.36 – 0.26	z = -2.37; p = 0.05	z = 2.71; p = 0.03	z = -0.52; p = 1.00	z = 0.21; p = 1.00
-0.2 – 0.42	z = -1.68; p = 0.32	z = 1.75; p = 0.32	z = -0.38; p = 1.00	z = 0.34; p = 1.00
-0.04 – 0.58	z = -0.94; p = 0.99	z = 1.16; p = 0.99	z = -1.1; p = 0.99	z = 0.9; p = 0.99
0.13 – 0.75	z = 0.65; p = 1.00	z = 0.15; p = 1.00	z = -1.27; p = 0.81	z = 0.46; p = 1.00
0.29 – 0.91	z = 1.13; p = 1.00	z = -0.77; p = 1.00	z = -0.83; p = 1.00	z = 0.4; p = 1.00
0.45 – 1.07	z = 1.84; p = 0.26	z = -0.65; p = 1.00	z = -0.83; p = 1.00	z = -0.43; p = 1.00
0.62 – 1.24	z = 3.37; p < 0.01	z = -1.35; p = 0.35	z = -1.66; p = 0.29	z = -0.38; p = 0.70
0.78 – 1.4	z = 4.29; p < 0.01	z = -2.17; p = 0.09	z = -1.92; p = 0.11	z = -0.28; p = 0.78
0.94 – 1.56	z = 4.4; p < 0.01	z = -1.27; p = 0.41	z = -2.86; p = 0.01	z = -0.3; p = 0.76
1.11 – 1.73	z = 4.32; p < 0.01	z = -0.79; p = 0.86	z = -3.79; p < 0.01	z = 0.22; p = 0.86
1.27 – 1.89	z = 4.33; p < 0.01	z = -1.08; p = 0.30	z = -4.75; p < 0.01	z = 1.43; p = 0.30

Trait Combination (column header spanning trait columns)

Supplementary References

1. Lima-Ribeiro, M. S. *et al.* EcoClimate: a database of climate data from multiple models for past, present, and future for macroecologists and biogeographers. *Biodiversity Informatics* **10**, 1–21 (2015).

2. Bennett, P. M. & Harvey, P. H. Relative brain size and ecology in birds. *Journal of Zoology* **207**, 151–169 (1985).

3. Jetz, W., Thomas, G. H., Joy, J. B., Hartmann, K. & Mooers, A. O. The global diversity of birds in space and time. *Nature* **491**, 444–448 (2012).
