# Supplementary material for: Alternative ecological strategies lead to avian brain size bimodality in variable habitats
Source: Nat Commun. 2019 Aug 23;10:3818. doi: 10.1038/s41467-019-11757-x (PMC6707158; doi:10.1038/s41467-019-11757-x)
Supplement: Supplementary file 3 — Reporting Summary [file 41467_2019_11757_MOESM3_ESM.pdf]

nature research

Corresponding author(s): DBPR

Last updated by author(s): Jun 16, 2019

Reporting Summary

Nature Research wishes to improve the reproducibility of the work that we publish. This form provides structure for consistency and transparency in reporting. For further information on Nature Research policies, see Authors & Referees and the Editorial Policy Checklist.

Statistics

For all statistical analyses, confirm that the following items are present in the figure legend, table legend, main text, or Methods section.

n/a	Confirmed	
☐	☒	The exact sample size (n) for each experimental group/condition, given as a discrete number and unit of measurement
☒	☐	A statement on whether measurements were taken from distinct samples or whether the same sample was measured repeatedly
☐	☒	The statistical test(s) used AND whether they are one- or two-sided<br>*Only common tests should be described solely by name; describe more complex techniques in the Methods section.*
☐	☒	A description of all covariates tested
☐	☒	A description of any assumptions or corrections, such as tests of normality and adjustment for multiple comparisons
☐	☒	A full description of the statistical parameters including central tendency (e.g. means) or other basic estimates (e.g. regression coefficient) AND variation (e.g. standard deviation) or associated estimates of uncertainty (e.g. confidence intervals)
☐	☒	For null hypothesis testing, the test statistic (e.g. F, t, r) with confidence intervals, effect sizes, degrees of freedom and P value noted<br>*Give P values as exact values whenever suitable.*
☒	☐	For Bayesian analysis, information on the choice of priors and Markov chain Monte Carlo settings
☒	☐	For hierarchical and complex designs, identification of the appropriate level for tests and full reporting of outcomes
☒	☐	Estimates of effect sizes (e.g. Cohen's d, Pearson's r), indicating how they were calculated

Our web collection on statistics for biologists contains articles on many of the points above.

Software and code

Policy information about availability of computer code

Data collection	Data was compiled in R 3.4.1
Data analysis	All analyses were performed in R 3.4.1 and relevant R code is included in Supplementary Data 1.

For manuscripts utilizing custom algorithms or software that are central to the research but not yet described in published literature, software must be made available to editors/reviewers. We strongly encourage code deposition in a community repository (e.g. GitHub). See the Nature Research guidelines for submitting code & software for further information.

Data

Policy information about availability of data

All manuscripts must include a data availability statement. This statement should provide the following information, where applicable:

- Accession codes, unique identifiers, or web links for publicly available datasets
- A list of figures that have associated raw data
- A description of any restrictions on data availability

Raw data used in analyses is included in Supplementary Data 2 and 3. R code used for analyses is included in Supplementary Data 1.

Field-specific reporting

Please select the one below that is the best fit for your research. If you are not sure, read the appropriate sections before making your selection.

☐ Life sciences ☐ Behavioural & social sciences ☒ Ecological, evolutionary & environmental sciences

For a reference copy of the document with all sections, see nature.com/documents/nr-reporting-summary-flat.pdf

Ecological, evolutionary & environmental sciences study design

All studies must disclose on these points even when the disclosure is negative.

Study description	We use randomization tests and quantile regression to analyze the relationship between brain size, environmental conditions , and additional ecological and life history traits for 2062 globally distributed species of birds.
Research sample	Our analyses include all avian species with available data (from published sources) on brain size (collated from published sources or measured from museum specimens in Fristoe et al. 2017), distribution (range maps from BirdLife International, 2015), diet (compiled from published sources by Wilman et al. 2014), reproductive output, and body size (both compiled from published sources by Myhrvold et al. 2015; see paper for full citations).
Sampling strategy	We analyzed all species with data available from the sources outlined in the 'Research sample' section and in the methods of the paper.
Data collection	Data was downloaded from published sources (see section 'Research sample' and citations in the manuscript). We extracted data from the datasets on distribution, reproductive output, body size, and diet for all species included in the brain size dataset. In cases where species names did not match, we consulted a list of synonyms included with Myhrvold et al 2015.
Timing and spatial scale	Species included in our analyses are globally distributed. All data used in analyses was downloaded between 2014 and 2017.
Data exclusions	We used brain size data for pelagic species when calculating relative brain size values, but excluded these species from subsequent analyses because the environmental measures that we used are unlikely to reflect the conditions that species that spend much of their time at sea typically encounter. We excluded data on reproductive output for Rhea americana and Alectura lathami because the exceptionally high values of clutch side included in Myhrvold et al 2015 were inconsistent with other sources.
Reproducibility	All data sources are cited in the paper and whenever possible, raw data was included in Supplementary Data 2. The code used to perform analyses is included in Supplementary Data 1.
Randomization	Randomizations are not applicable because we did not perform experiments. Our data was compiled from published research.
Blinding	Blinding is not relevant to our research because we did not perform experiments. Our data was compiled from published research.

Did the study involve field work? ☐ Yes ☒ No

Reporting for specific materials, systems and methods

We require information from authors about some types of materials, experimental systems and methods used in many studies. Here, indicate whether each material, system or method listed is relevant to your study. If you are not sure if a list item applies to your research, read the appropriate section before selecting a response.

Materials & experimental systems

n/a	Involved in the study
☒	☐ Antibodies
☒	☐ Eukaryotic cell lines
☒	☐ Palaeontology
☒	☐ Animals and other organisms
☒	☐ Human research participants
☒	☐ Clinical data

Methods

n/a	Involved in the study
☒	☐ ChIP-seq
☒	☐ Flow cytometry
☒	☐ MRI-based neuroimaging
