# Supplementary material for: Alternative ecological strategies lead to avian brain size bimodality in variable habitats
Source: Nat Commun. 2019 Aug 23;10:3818. doi: 10.1038/s41467-019-11757-x (PMC6707158; doi:10.1038/s41467-019-11757-x)
Supplement: Supplementary file 4 — Description of Additional Supplementary Files [file 41467_2019_11757_MOESM4_ESM.pdf]

Description of Additional Supplementary Files

File Name: Supplementary Data 1
Description: R code required to rerun all presented analyses.

File Name: Supplementary Data 2
Description: Table including data for all species used in our analyses. See the Methods Section of the main text details on how variables were calculated and for original data sources.

File Name: Supplementary Data 3
Description: Rdata file containing global rasters of environmental PC1 and PC2 (see Methods), as well as results from the principal component analysis used to produce them (requires the R packa ge 'psych').
